# Metal versus Fiberglass Post-Orthodontic Retainers Short-Term Effects on Plaque Index and Microbial Colonization: An Observational Study

**DOI:** 10.3390/life12030331

**Published:** 2022-02-23

**Authors:** Stefano Mummolo, Vincenzo Quinzi, Alessandro Nota, Carla Marino, Laura Pittari, Rebecca Jewel Manenti, Simona Tecco

**Affiliations:** 1 Department of Life, Health and Environmental Sciences, University of L’Aquila, 67100 L’Aquila, Italy; stefano.mummolo@univaq.it (S.M.); vincenzo.quinzi@univaq.it (V.Q.); carla.marino@univaq.it (C.M.); rebeccajmanenti@univaq.it (R.J.M.); 2 Dental School, Vita-Salute San Raffaele University and IRCCS, San Raffaele Hospital, 20132 Milan, Italy; nota.alessandro@hsr.it (A.N.); laura_pittari@hotmail.it (L.P.)

**Keywords:** orthodontic materials, metal, fiberglass, post-orthodontic retainer, interceptive orthodontics, streptococcus mutans, lactobacilli, orthodontic fixed appliances, pediatric dentistry

## Abstract

In orthodontics, post-treatment retention phase is crucial for maintaining the obtained clinical results. In cases of crowding, a bonded fixed retainer is often chosen to maintain teeth alignment in the anterior sector of the lower dental arch. A fixed retainer can remain in the mouth for years. Therefore, it is important that it is applied with harmless materials for the level of plaque control. The present study aimed to investigate the salivary concentrations of Streptococcus mutans (*S. mutans*) and *Lactobacilli*, and the Sillness and Loe plaque index, in patients wearing metal wire versus fiberglass orthodontic retainers. Forty post-orthodontic patients were included in the sample: in 20 subjects a metal wire retainer was applied (MR), while in the others a fiberglass retainer was applied (FR). The variables were recorded at baseline (T0), after 1 month (T1), and after 2 months (T2) of follow-up. The percentage of patients with a level of *S. mutans* and *Lactobacilli* colonization > 10^5^ increased over time in the FR group (T0 = 0%, T1 = 5%, T2 = 35%), compared with the MR group. PI increased in the FR group (T0 = 0, T1 = 14, T2 = 27), and remained almost the same in the MR group (T0 = 3, T1 = 0, T2 = 2). From the present results it appears that the metal wire retainer is better than the fiberglass retainer for the level of plaque control performed by the patients.

## 1. Introduction

In orthodontics, post-treatment retention phase is crucial for maintaining the obtained clinical results [1]. In cases of crowding, a bonded fixed retainer is often chosen to maintain teeth alignment in the anterior sector of the lower dental arch.

A fixed retainer can remain in the mouth for years [1]. Therefore, it is important that it is applied with harmless materials. Nowadays the most used materials are metal wire and fiberglass [2]. However, few studies have been published on its effect on bacterial flora, and on the level of plaque control performed by the patients [3,4,5,6]. 

The great part of the studies compared fixed and removable retainers, concluding that they do not differ in salivary Streptococcus mutans and Lactobacillus casei levels and plaque index [3], although Gökçe B et al. (2019) [4] observed that gingival health improves with Essix removable retainer, but not with fixed metal retainers. In comparison with control subjects without any retainer, [5] it appeared no difference was observed in the status of the periodontal tissue between individuals with and without fixed metal retainers. Among metal wire fixed retainers more plaque was observed on the distal surfaces of the lower anterior teeth in subjects with multistrand wire retainers than in subjects with round wire retainers [6]. Recently, Sinha et al. (2021) compared metal wire retainer to ceramic retainer and concluded that metal wire results associated to a higher plaque index during six months [7].

Fiberglass and metal wire retainers showed similar results in terms of bond failure and breakage after 6 years of retention [8]. But they have never yet been compared with regard to the level of bacterial plaque accumulation and the level of bacterial colonization. And no comparison was performed between them for the level of plaque control performed by the patients. Bacterial concentration can affect carioreceptivity, the predisposition of an individual to be affected by carious pathology. Consequently, bacterial concentration can be useful to diagnose individual carioreceptivity [9,10,11,12,13].

One method to evaluate bacterial concentration in the saliva is represented by in-office salivary tests. Through these tests the clinician can study all the characteristics of saliva. Generally, a salivary test allows the clinician to highlight a few bacterial species, present in a state of equilibrium. Tooth decay occurs when this balance is altered. The bacterial species responsible for caries are Streptococcus mutans and *Lactobacilli*, which with their metabolism, in the presence of sugars, produce acids that lower the environmental pH (pH < 4.8) causing damage to the hard tissues of the teeth, i.e., caries.

In-office salivary tests allow to establish the level of bacterial colonization in saliva; if this level exceeds some limits, we speak of a high risk of caries (high carioreceptivity), therefore, thanks to these tests, we can define the patient predisposed to the onset of caries.

The presence of *Lactobacilli* in saliva in quantities exceeding the norm indicates a risk factor for the patient, due to an excessively rich diet of sugars, accompanied by poor oral hygiene [12,13]. On the other hands, an excessive presence of Streptococci indicates the presence of plaque in quantities greater than the norm, therefore indicating an individual predisposition and/or poor oral hygiene.

During the post-orthodontic period, if the patient wears fixed retainers, it becomes essential to monitor oral health, and rapid salivary tests can define carioreceptivity. It is essential to define the colonization of Streptococcus mutans, but this is not enough, as it is also crucial to investigate the presence of *Lactobacilli*, which, moreover, can also be present alone [3,4].

Thus, the aim of the present study was to compare the effect of metal wire retainers (MR) versus fiberglass retainers (FR) on the salivary concentration of *S. mutans* and *Lactobacilli* in post-orthodontic patients, and on plaque index [5]. The null hypothesis is that there is no difference between the retainers for the level of plaque control performed by the patients.

## 2. Experimental Section

The protocol was approved by the Ethic Committee of the University of L’Aquila (Italy) (protocol code DR 206/2013, dated 10 January 2014) The patients were informed about the purposes and methods of the study, and their signed informed consents were obtained.

For the numerosity of the samples, we applied the formula [9]:

N = t^2^ P (1 − P)/α^2^

where N is the numerosity of the sample, t is the t distribution; P is the expected prevalence (in this case 10%). We considered a normal value of *S. mutans* as <10^5^ and a mean difference of approximately 10% level as clinically significant difference between the study and the control group, in the prevalence of subjects with *S. mutans* counts > 10^5^. The same difference was also applied to the *Lactobacillus* spp. counts. Therefore considering α = 0.06, the sample size requested was 20 subjects.

A sample of 40 post-orthodontic patients aged between 28 and 33 years was enrolled in the study. Initially, adult subjects with orthodontic devices were individuated in our clinic. Then 40 subjects without clinical situation who could have affected the plaque accumulation in the anterior areas (as for example, bone fenestration, due to a particular clinical malocclusion) were included in the final sample. Also, subjects with diagnosed periodontal pathology with loss of periodontal tissue were excluded. Then a simple randomization was applied.

In twenty subjects post orthodontic fixed retainers in metal wire were applied (group MR), and in the other 20 patients retainers in fiberglass were applied (group FR).

When the orthodontic treatment was completed, fixed orthodontic vestibular appliances were removed and professional oral hygiene with scaling and polishing of dental arches was performed. Then, the retainers were all applied by the same operator (one of the authors)

**Figure 1:**
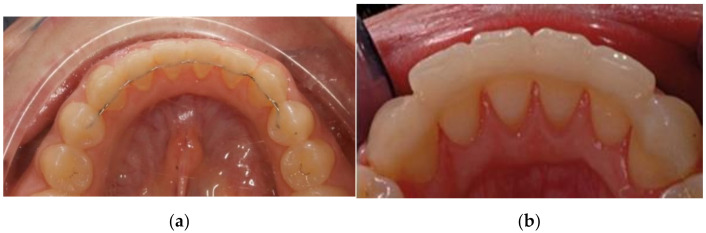
Metal wire retainer (**a**) and fiberglass retainer (**b**).

In groups MR, the lingual surface of six/eight lower teeth was firstly etched by 37% of phosphoric acid gel (3M Unitek, Monrovia, CA, USA) for 30 s. Metal wire retainer was made with the 0.0175 stainless steel twisted wire (G&H Orthodontics, Franklin, IN, USA). After rinsing with water and air drying, the retainers were held still, resting on the lingual surfaces of the teeth with loops of dental floss placed in the interproximal spaces. Then, the retainers were fixed with a thin layer of bonding primer (Transbond XT, 3M Unitek, Monrovia, CA, USA). Then, composite resins were light-cured and polished carefully (Figure 1a).

Subjects in group FR received fiber reinforced composite retainers (Fiber Splint, 2 mm, Polydentia s.a., Mezzovico-Vira, Switzerland). After isolation of lower anterior teeth, dental floss was used for distance measurement, and an adequate length of ribbon fiber was cut. It was pretreated with adhesive primer (3M ESPE). The lingual surface of six/eight lower teeth was firstly etched by 37% of phosphoric acid gel (3M Unitek, Monrovia, CA, USA) for 30 s. After rinsing with water and air drying, a thin layer of adhesive primer (3M ESPE) was [2] applied, and light cured for 15 s (Otholux; 3M) which was followed by the application of flowable composite resin (3M ESPE). Then fiber ribbon was adapted with plastic instrument, surplus composite was cleared, and each tooth was light cured for 15 s (Figure 1b).

All the visits were conducted at the University of L’Aquila. The research protocol was carried out by performing in-office salivary tests in three different sessions, each 30 days apart. The professional oral hygiene procedures performed in association with the retainer application had the aim of reducing the bacterial concentration and the dental plaque after the orthodontic treatment as it was observed that during fixed orthodontic treatment there is an alteration of the bacterial flora [9].

The day after the application of the retainer and the associated oral hygiene procedures (T0) the Sillness and Loe plaque index (PI) from lower canine/first bicuspid to canine/first bicuspid (six sites for each tooth) was assessed, and the in-office salivary test was performed to calculate the bacterial count of *S. mutans* and *Lactobacilli*. After 30 days (T1) and after 60 days (T2), the same variables were re-evaluated. All the data were recorded by the same expert operator (S.M.).

### 2.1. In-Office Salivary Test

In the present investigation, the in-office salivary test Ivoclar© (Ivoclar Vivadent s.r.l., Bologna, Italy) was used to evaluate the microbial count [9,10,11].

Whole stimulated saliva was collected from each patient. Patients were asked not to eat, drink, or brush their teeth for at least one hour prior to testing, as required by the manufacturer, as these activities can affect salivary flow.

At each appointment, saliva testing was performed prior to the clinic visit. The patient was asked to chew a paraffin tablet for 30 s and then collect the saliva in a shot glass [9].

For each test, a small amount of saliva was taken from the small glass through a pipette. *S. mutans* colonies were detected as small blue colonies with a diameter of <1 mm on the blue agar, while Lactobacillus colonies were detected as white colonies on the clear agar [9,10,11]. Comparison with the corresponding pictures in the model chart permitted the assessment of the caries risk. Findings of 10^5^ CFU or more of *Lactobacilli* and *S. mutans* per mL saliva indicated a high caries risk [9,10,11,12].

### 2.2. Data Treatment

In each group, Plaque index (PI) was handled as mean and standard deviation. Differences between the two groups were calculated with Students’ test for independent samples. 

Intra-group differences were tested with ANOVA statistic for paired samples.

The bacterial colonization was calculated for each group as the number of subjects with *S. mutans* or *Lactobacilli* colonization > 10^5^ CFU for mL of saliva. Percentages in the two groups were compared with the Chi-square test.

For each analysis, the *p*-value was set at the 0.05 level. Statistical analysis was performed with StatPlus Pro for MAC (build 7.3.3.0/Core v7.3.32; AnalystSoft Inc., 2020, Walnut, CA, USA).

## 3. Results

Table 1 shows demographic data and salivary parameters at the beginning of the study (T0) in both groups. There was no significant difference between the two groups at T0.

Table 2 indicates mean and standard deviation of PI for each group over time, and the comparison between the two groups. The mean PI value at T2 in the MR group is significantly lower than the results in the FR group.

During the follow-up, no critical factor for the appearance of plaque was detected: no breakage of the devices, or other relevant clinical situations, were recorded for any of the patients.

Table 3 shows the percentage of patients with *S. mutans* > 10^5^ CFU and the comparison between the two groups. The amount of bacteria decreased over time in the MR group; while in the FR group it progressively increased.

Table 4 indicates the percentage of patients with Lactobacillus >10^5^ CFU and the comparison between the two groups. In this case, the amount of bacteria in the MR group decreases at T1 (after 30 days), but increases at T2 (after 60 days). However, the average values of the MR group are lower than the values of the FR group, in which the amount of the bacteria increases over time.

## 4. Discussion

From the present analyses, it emerged that the two materials (metal wire and fiber glass) show specific influences on the level of plaque control performed by the patients, as assessed also by previous literature [13,14,15]. Specifically, it was seen a much lower amount of plaque in the MR group, with respect to the FR group, at T1 and T2.

This difference is certainly correlated to the different material because the retainers were monitored for bonding failure during the whole follow-up, and no clinically detectable changes were recorded for any of the retainers during follow-up, which could involve or explain the detected changes in salivary indices. The absence of any incidents is reasonably justified considering that the follow-up period of the present study was rather short. A short-term follow-up was preferred to a long-term follow-up because the primary aim of the study was to investigate a possible difference between the two different materials. With a longer follow-up, other conditions (such as, for example, bonding failure or poor oral hygiene) could have explained the observed changes in the saliva. In the previous literature, increases in plaque were related to the time that the retainers were in place rather than on the type of wire [11]. Maybe, the FR retainer, due to its greater width of 2 mm compared to MR, covers a greater surface of tooth, resulting in increased area for possible accumulation of bacterial plaque. In addition, in a previous study, it was reported much higher amount of plaque on the distal surfaces of the lower anterior teeth in the group with multistrand wire retainers; the present findings do not confirm that observation, probably associated to the distal crease modeled on the metal wires [6], that was not performed in the present cases. In addition, a previous study with SEM analysis evidenced that fiberglass retainers show structural characteristics that could explain microbial accumulation: differences in dimension, number, diameter and orientation of fibers, mostly when the retainer is applied without a preliminary treatment through impregnation of the fibers with fluid resin, give a complex structure that makes it less resistant to bacterial agents [16].

The present findings also evidence the colonization of *S. mutans* and *Lactobacilli* [17], because a progressive increase in bacterial colonies emerged over time in the FR group, with respect to the MR group, in which a decrease was appreciated [18].

At the present, these data suggest preferring metal material for orthodontic retainers with respect to fiberglass, for the level of plaque control performed by the patients, and for microbiological colonization. It can be deduced that metal material respects the salivary characteristics more than fiberglass [19], also considering that the metal retainer seems better wear-resistant to the daily brushing procedure during home oral hygiene and that wear can reduce the resistance of the material, change its mechanical properties, and lead to bacterial adhesion [12].

A previous in-vitro evaluation also evidenced that fiber bundle is attacked by acids potentially present in the oral cavity; the degree of aggressiveness depended on the acid concentration, thus suggesting preserving a careful plaque control is necessary, especially in the interproximal spaces, to avoid acid formation [20].

Thus, in daily practice, the primary advantage of FR splints over conventional MR is aesthetics. Fibers are barely invisible and do not affect the translucency of teeth. This aspect seems important, mostly considering the higher number of adult patients who request an orthodontic therapy. Then, FR are metal-free and are indicated for adult and young patients screened by Nuclear Magnetic Resonance or in subjects allergic to metals [21,22,23].

The present findings agree with those observed during an orthodontic treatment with a fixed orthodontic metal appliance (the palatal expander) [24]. Differently, silicon-removable appliances evidenced a negligible effect on the same salivary indices [25].

From a clinical point of view, the present data are much more relevant than those collected with other orthodontic appliances, because the retainer remains in the oral cavity for many years and should affect the carioreceptivity as little as possible. Thus, all data on its biocompatibility, and its effect on the oral microbiota are clinically useful for the management of this type of therapy, as well as for all dental materials destined to remain in the mouth for a long time [4,26].

Compared to the other studies where periodontal health was evaluated only clinically, the present methodology, including the assessment of bacterial contamination in saliva, suggests the clinician to be guided by in-office salivary tests in identifying the proper hygiene follow-up protocol based on the type of retainer.

This study shows some limitations, as there was no stratified randomization in the allocation of patients. In addition, it cannot be excluded that some systemic condition (as, for example, pre-treatment position of teeth) [27], or systemic predisposition of the patient [28], or a lack of collaboration could have influenced the result of the present research. In addition, a greater sample could have allowed us to evaluate other variables, crucial to define periodontal health (for example, bleeding index, probe depth, salivary flow, and saliva buffer capacity).

Future studies will be aimed to include in a greater sample size, and stratify the specimen based on the initial malocclusion and initial periodontal status.

Considering the short follow-up of the present sample, that is a limitation, it would be important and auspicious in future to extend the research over time, to ensure that orthodontists can advise and follow their patients, preserving them from the risk of incurring in unnecessary periodontal or caries problems after orthodontic treatment [29].

## 5. Conclusions

From the present analyses, it emerged that the two materials (metal wire and fiber glass) show specific influences for the level of plaque control performed by the patients. It appears that metal wire retainer is better than the fiberglass retainer. In addition, *S. mutans* and *Lactobacilli* colonization increased over time in subjects with the fiberglass retainer, with respect to subjects with the metal-wire retainer. Future studies should be carried out to extend the follow-up over time in a greater sample.

## Figures and Tables

**Table 1:** Demographic data and plaque index at T0 in the MR and FR groups.

	MR Group (n = 20)	FR Group (n = 20)	Between Groups Differences *
Age range (years)	30.2 ± 2.4	30.75 ± 2.2	t = −0.84; *p* = 0.403 (n.s.)
PI (T0)	0.5 ± 0.37	0.45 ± 0.26	t = −0.28; *p* = 0.779 (n.s.)

* calculated by using Students’ test for independent samples. n.s. = not significant.

**Table 2:** Plaque indices (PI) overtime in the two groups, and between groups comparisons at each time.

	PI (T0)	PI (T1)	PI (T2)	Intra-Group Difference
MR group	0.5 ± 0.37	0.75 ± 0.51	0.7 ± 0.43	F = 0.108; *p* = 0.897 (n.s.)
FR group	0.45 ± 0.26	0.65 ± 0.56	1.7 ± 0.3	F = 6.047; *p* = 0.004 n.s.
Between groups difference	t = −0.28; *p* = 0.779 (n.s.)	t = 0.43; *p* = 0.667 (n.s.)	t = −4.81; *p* < 0.001	

**Table 3:** Percentage of patients with *S. mutans* colonies >10^5^ CFU over time in the two groups.

Percentage of Patients with *S. mutans* >10^5^ CFU	T0	T1	T2	T0 vs. T1	T0 vs. T2	T1 vs. T2
MR group	10%	0%	0%	NS	NS	NS
FR group	0%	5%	35%	NS	Chi-square = 9.37; *p* = 0.002	Chi-square = 36.5 *p* = 0.001

**Table 4:** Percentage of patients with *Lactobacilli* colonies >10^5^ CFU over time in the two groups, and between groups comparisons at each time.

Percentage of Patients with Lactob. > 10^5^ CFU	T0	T1	T2	T0 vs. T1	T0 vs. T2	T1 vs. T2
MR group	15%	0%	10%	NS	NS	NS
FR group	0%	5%	35%	NS	Chi-square = 24.967; *p* = 0.00	NS

## Data Availability

The data that support the findings of this study are available from the University of L’Aquila, but restrictions apply to the availability of these data, which were used under license for the current study and so are not publicly available. Data are, however, available from the authors upon reasonable request and with permission of the University of L’Aquila partner.
